# Signal-Amplified Lateral Flow Test Strip for Visual Detection of Cu^2+^

**DOI:** 10.1371/journal.pone.0169345

**Published:** 2017-01-10

**Authors:** Yulong Wang, Limin Wang, Juanjuan Xue, Jinbo Dong, Jia Cai, Xiude Hua, Minghua Wang, Cunzheng Zhang, Fengquan Liu

**Affiliations:** 1 Institute of Plant Protection, Jiangsu Academy of Agricultural Science, College of Plant Protection, Nanjing Agricultural University, Nanjing, P. R. China; 2 College of Plant Protection (Key Laboratory of Integrated Management of Crop Diseases and Pests), Nanjing Agricultural University, Nanjing, P. R. China; 3 Institute of Food Quality Safety and Detection Research, Jiangsu Academy of Agricultural Sciences, Nanjing, P. R. China; New York University, UNITED STATES

## Abstract

A signal-amplified lateral flow test strip (SA-LFTS) for the detection of Cu^2+^ in aqueous solution was constructed based on Cu^+^-catalyzed click chemistry and hybridization of single-stranded DNA (ssDNA). Alkyne and azide modified ssDNA acted as specific elements for Cu^2+^ recognition, and a chemical ligation product formed through Cu^+^-catalyzed alkyne–azide cycloaddition. Hybridization of ssDNA-labeled gold nanoparticles resulted in high sensitivity, and the output signal could be observed directly by the naked eye. Using the developed SA-LFTS under optimal conditions, Cu^2+^ could be detected rapidly with limit of detections of 5 nM and 4.2 nM by visual observation and quantitative analysis, respectively. The sensitivity (i.e. the visual limit of detection) of the SA-LFTS was 80-times higher than that of traditional LFTS. The SA-LFTS was applied to the determination of Cu^2+^ in municipal water and river water samples with the results showing good recovery and accuracy. The developed test strip is promising for point-of-care applications and detection of Cu^2+^ in the field.

## Introduction

Copper (Cu^2+^) is an essential trace element, and has important roles in various physiological processes [1]. However, long-term exposure to high levels of Cu^2+^-based toxins is a known health hazard and can cause gastrointestinal distress [2], liver or kidney damage [3], and various neurological diseases [4, 5]. Analytical techniques, such as inductively coupled plasma mass spectroscopy [6, 7], atomic absorption spectroscopy [8], colorimetry [9] and voltammetry [10] are routinely used for Cu^2+^ analysis. However, these analytical techniques offer high sensitivity and accuracy, they require expensive and sophisticated instruments and skilled personnel.

Recently, functional nucleic acid-based sensors have shown potential for detection of Cu^2+^. Among these sensors, DNAzyme sensors are based on Cu^2+^-dependent DNA cleavage, where Cu^2+^ functions as a cofactor for hydrolytic cleavage of a ribonucleotide linkage in the substrate sequence of a DNAzyme/substrate complex [11–13]. However, applications of DNAzyme sensors are limited because the activity of DNAzyme is affected by the concentration of salts, temperature, and pH. Cu^+^-catalyzed alkyne–azide cycloaddition reaction, a click chemistry reaction, involves an alkyne and an azide group reacting to form a 1,2,3-triazole under Cu^+^ catalysis [14]. This reaction has attracted much attention because of its high yield and high selectivity. A trace amount of Cu^+^, generated from the reduction of Cu^2+^ in the presence of sodium ascorbate, is required as a catalyst for efficient conjugation between azide and alkyne groups, and this provides an important copper-mediated signal for Cu^2+^ detection [15]. This reaction can tolerate a wide range of solvents, ionic strengths, temperatures, and pH values, enabling robust sensing under different conditions [16, 17]. Several biomolecule methods for Cu^2+^ detection based on Cu^+^-catalyzed azide−alkyne cycloaddition have been reported. However, some of these methods are based on conventional organic reactions and have long reaction times (~24 h) [15, 18] or need washing steps to reduce the background signal, both of which complicate the assay procedure. Moreover, most of these methods are not suitable for in-situ or real-time detection.

Lateral flow test strips (LFTS) are powerful tools for on-site screening, and have attracted increasing attention in clinical diagnosis because of their simplicity, portability, long-term stability, and low cost. Wang et al. have successfully developed a strip biosensor for the detection of Cu^2+^ based on click chemistry, but with less sensitivity[19]. Compared to LFTS the signal amplified lateral flow test strip (SA-LAFTS) with a second amplification pad for single-stranded DNA hybridization shows excellent signal amplification performance [20]. This method uses the conjugation of two ssDNA labeled gold nanoparticle (AuNP) probes to increase the number of AuNPs accumulated on the test line, which increases the sensitivity sufficiently to meet the detection requirements for particular target analytes even at trace concentrations.

The aim of the present study was to develop a SA-LFTS for rapid and more sensitive detection of Cu^2+^ based on Cu^+^-catalyzed click chemistry and ssDNA hybridization. In the presence of Cu^2+^ and a reductant (sodium ascorbate), Cu^+^-catalyzed azide-alkyne cycloaddition would result in chemical ligation of ssDNA modified with alkyne and azide groups. The generated click-ligated long chain ssDNA could be applied to test strip. Compared to earlier reports, the method showed higher sensitivity and excellent selectivity for the detection of Cu^2+^ within 40 min, and the procedure could be carried out on-site with detection by the naked eye. Rapid Cu^2+^ detection in municipal water and river water samples was also demonstrated.

## Materials and Methods

### Reagents and apparatus

Sodium ascorbate, tris-(hydroxypropyltriazolylmethyl) amine (THPTA), Tween-20, bovine serum albumin, sodium dodecyl sulfate (SDS), CuCl_2_^.^2H_2_O, and other metal salts were purchased from Sigma-Aldrich (St. Louis, MO, USA). AuNPs (20 nm 10 nM) were supplied by Jiening Biotech Co., Ltd. (Shanghai, China). The elements to prepare the signal amplified lateral flow test strips, including nitrocellulose membranes, glass fibers, and absorbent pads, were purchased from Millipore Corp. (Bedford, MA, USA). Other reagents and chemicals were of analytical grade and used without purification.

Streptavidin and oligonucleotides purified by HPLC were supplied by Shanghai Sangon Biotechnology Co., Ltd. (Shanghai, China). DNA-A and biotinylated DNA-B, which have an azide group and an alkyne group, respectively, were used as Cu^2+^-mediated reagents. Biotinylated DNA-C was used to prepare the probe on the control line. Signal probes were prepared with AuNPs and capture strands (S1) and signal amplification strands (S2) for DNA-AuNPs probe 1, or enhancement strands (S3) for DNA-AuNPs probe 2). These sequences of the oligonucleotides were listed as follows:

DNA-A: 5’-azide-TAGTCTGATTGC-3’;
DNA-B: 5’-biotin-ATCCTTATCAAT-alkyne-3’;
S1: 5’-thiol-GGCCGGGCAATCAGACTA-3’;
S2: 5’-thiol-CCGGCCACACAGATACTC-3’;
S3: 5’-thiol-CCGGCCGAGTATCTGTGT-3’;
DNA-C: 5’-biotin-CCGGCCTAGTCTGATTGC-3’.

Municipal water samples were collected from our laboratory and river water samples (Xuanwu Lake, Nanjing, China) were filtered with 0.45 μm cellulose acetate filters.

All aqueous solutions were prepared with ultrapure water (18.2 MΩ cm^–1^) from a Millipore Milli-Q water purification system (Millipore Corp.). An XYZ 3060 dispensing platform and CM4000 Guillotine Cutter (BioDot, Irvine, CA) were used to prepare test strips.

### Preparation of the DNA-AuNPs probes

The DNA-AuNPs probe 1 conjugate was prepared as described previously [21] with slight modification. Briefly, 45 μL of 1 OD thiol-modified capture strands (S1) and 135 μL of 1 OD signal amplification strands (S2) were added to 900 μL of a concentrated solution of AuNPs (10-fold; 100 nM) and stirred for 24 h at room temperature (RT). The DNA-AuNPs conjugates were aged by adding NaCl up to a final concentration of 300 mM, and stirred for another 24 h at RT. Then, a 1% sodium dodecyl sulfate (SDS) solution was added to reach a final SDS concentration of 0.01%. The solution was allowed to stand at RT for 4 h. The conjugates were washed by centrifugation at 12,000 ×*g* for 20 min, and rinsed with ultrapure water twice to remove excess DNA. The resulting solution was suspended in 200 μL of a buffer solution containing 20 mM Na_3_PO_4_, 5% bovine serum albumin, 0.25% Tween-20, and 10% sucrose. The preparation of DNA-AuNPs probe 2 conjugate was achieved in the same way using only enhancement strands (S3). All of the DNA-AuNPs probes were stored at 4°C until required.

### Preparation of the control probe

To facilitate immobilization of the streptavidin-biotinylated DNA-C on the control line, streptavidin was reacted with the biotinylated DNA-C. Briefly, 20 μL of 1 OD biotinylated DNA-C was mixed with 200 μL of 2 mg/mL streptavidin. After incubation for 1 h at RT, the excess DNA was removed by centrifugation at 6000 ×*g* for 30 min with a centrifugal filter (Cutoff 30000, Millipore). The conjugate was washed twice with 500 μL of 0.01 M phosphate-buffered saline (PBS). The resulting solution was suspended in 200 μL of 0.01 M PBS and stored at 4°C.

### Preparation of the SA-LFTS

The test strip consisted of the following five main elements: a sample pad, detection pad, amplification pad, nitrocellulose membrane, and an absorbent pad. The detection pad and the amplification pad were saturated with a membrane blocking solution (Invitrogen), and then dried at 37°C for 2 h. Then, DNA-AuNPs probe 1 and DNA-AuNPs probe 2 were dispensed onto the detection pad and amplification pad, respectively, using the XYZ-3060 dispensing platform, followed by drying at 42°C for 60 min. The test line and control line on the nitrocellulose membrane were prepared using 2 mg/mL streptavidin and 2 mg/mL control probe solutions, respectively, and then dried for 2 h at 37°C. All components were sequentially laminated onto a polyvinyl chloride backing card with 2 mm overlaps to ensure favorable migration of the test solution across the whole test strip. Finally, 5-mm wide LFTSs were cut from the prepared test strip and stored at 4°C.

### Visual detection of Cu^2+^

In a typical experiment, azide-modified DNA-A and alkyne-modified biotinylated DNA-B were mixed at a mole ratio of 1:1 in reaction buffer (0.01 M Tris-HCl containing 0.2 M NaCl, pH 7.0) to give a final concentration of 0.1 μM. Subsequently, 800 μM sodium ascorbate, 1 mM THPTA, which increases Cu^+^ binding, and various concentrations of Cu^2+^ (0–100 μM) were added. The mixture was incubated for 30 min at room temperature with shaking, and then a 20 μL aliquot was mixed with 80 μL of running buffer (4× saline sodium citrate containing 1% Tween-20) for application to the sample pad of the strip. The solution migrated toward the absorption pad because of capillary action. The test and control lines were evaluated visually within 10 min. For quantitative measurements, the relative intensity (T/C: optical intensity of the red band on the test line/control line) was recorded using a portable strip reader. A control experiment (i.e. in the absence of Cu^2+^) was conducted under the same conditions.

### Analysis of municipal water and river water samples

Municipal water and river water samples (Xuanwu Lake, Nanjing, China) were filtered through 0.45 μm cellulose acetate filters. The water samples were diluted two-fold with 20 mM Tris-HCl buffer containing 0.2 μM DNA-A and DNA-B. The pH and concentrations of sodium ascorbate (800 μM) and THPTA (1 mM) in each water sample were adjusted to be similar to those of the optimized working conditions. Different amounts of Cu^2+^ standard solution were spiked into the water samples. These mixtures were then analyzed with the proposed method and percent recovery values were calculated.

## Results and Discussion

### Principles of the SA-LFTS

**Fig 1:**
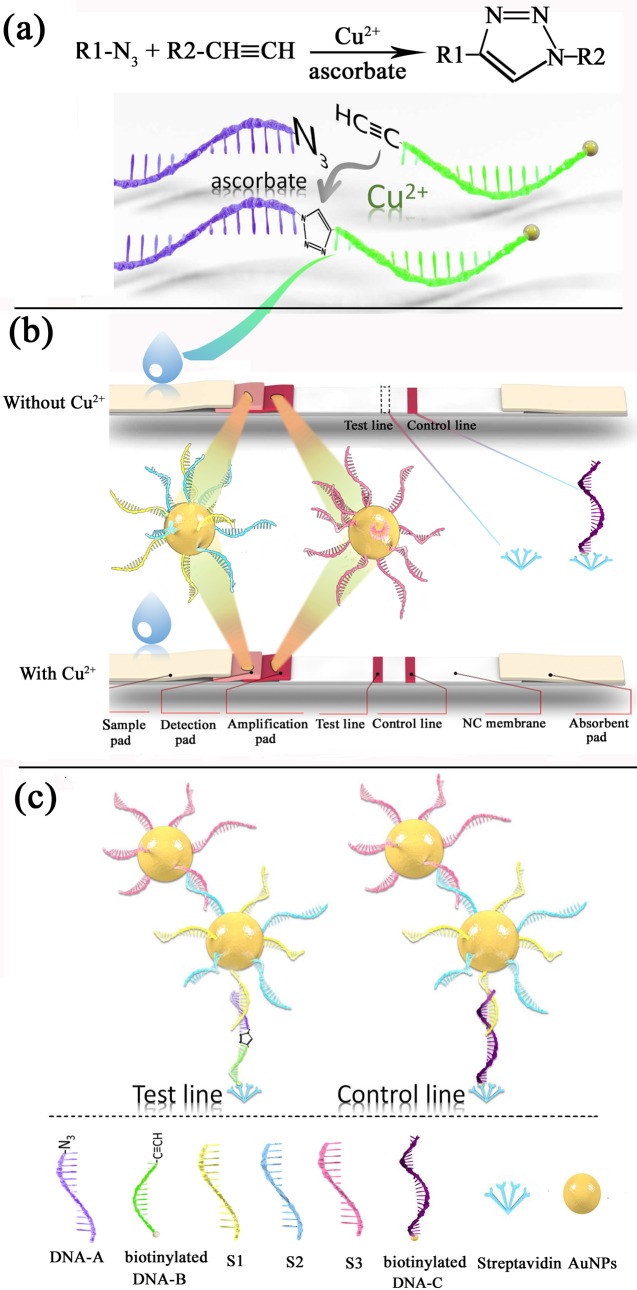
Lateral flow test strip. (a) Cu^2+^ detection based on Cu^+^-catalyzed click chemistry. (b) Schematic illustration of the SA-LFTS for the visual detection of the generated click-ligated ssDNA product. Compare with the traditional LFTS developed by Zeng’s group [19], the SA-LFTS contains an additional amplification pad, which results in higher sensitivity, and streptavidin-biotin interaction at the test line make the test be more easily implemented. (c) The test line and control line of the SA-LFTS after flow with Cu^2+^ addition. AuNPs are captured by streptavidin-biotin interaction and ssDNA hybridization.

The principles of the SA-LFTS for Cu^2+^ detection are shown in Fig 1. DNA-A and biotinylated DNA-B, which bear an azide group and an alkyne group, respectively, are used as Cu^2+^-mediated reagents. In the presence of Cu^2+^, a cycloaddition reaction between azide- and alkyne-functionalized ssDNA occurs to generate a click-ligated long chain ssDNA, (DNA-A–DNA-B) which is biotinylated, is applied to the test strip (Fig 1B). First, on the detection pad of the strip, the generated long chain ssDNA hybridizes with the DNA-AuNPs probe 1, which is partially complementary to the DNA-A. When this complex migrates to the test line, it is captured by streptavidin-biotin interaction for binding the AuNPs, and produces a color response. Excess DNA-AuNPs probe 1 is captured on the control line via hybridization between the DNA on probe 1 and the pre-immobilized control probe (DNA-C). A red band appears on the control line where the AuNPs-binds to DNA–C to confirm proper functioning of the test strip. The DNA-AuNPs probe 2 on the amplification pad then conjugates with the DNA-AuNPs probe 1 via hybridization between the two complementary ssDNA, and AuNPs accumulate on the test line (Fig 1C). In this way, the ssDNA-hybridization provides signal amplification. By monitoring the color change at the test line, we could qualitatively determine the concentration of the Cu^2+^. Quantitative analysis is performed by recording the relative intensity (T/C) with a portable strip reader.

### Ratio of S1 to S2 in DNA-AuNPs probe 1

**Fig 2:**
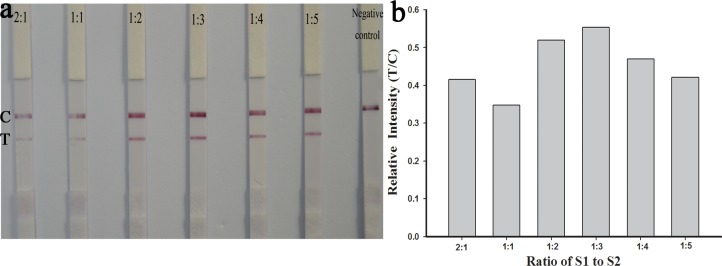
Sensing results for SA-LFTS with different ratios of capture strands (S1) to signal amplification strands (S2) in the DNA-AuNPs probe 1.

DNA-AuNPs were prepared by Au-SH conjugation reactions and S1 and S2 ssDNAs were used as capture and signal amplification strands, respectively. The probe’s functionality was based on the interaction of the ssDNA (S1 and S2) with AuNPs. The ratio of S1 and S2 is one of the most important parameters that affect the efficiency of target recognition and the signal amplification ability [22]. Different ratios of S1 to S2 (2:1, 1:1, 1:2, 1:3, 1:4 and 1:5) were tested. Relative intensity (T/C) was chosen as the evaluation criteria. The relative intensity (T/C) increased as the proportion of S2 in the S1:S2 mixture increased from 2:1 to 1:1, 1:2, and 1:3, and the S1:S2 mixture showed a modest maximum between 1:2 and 1:3 (Fig 2). At a S1:S2 ratio of 1:3, effective target capture and signal amplification were obtained. However, at ratios above 1:3, the signal amplification was achieved at the expense of target capture efficiency, which ultimately restricted the amplification of signal intensity. The optimal ratio of S1 to S2 was 1:3.

### Concentrations of the alkyne and azide modified ssDNA

**Fig 3:**
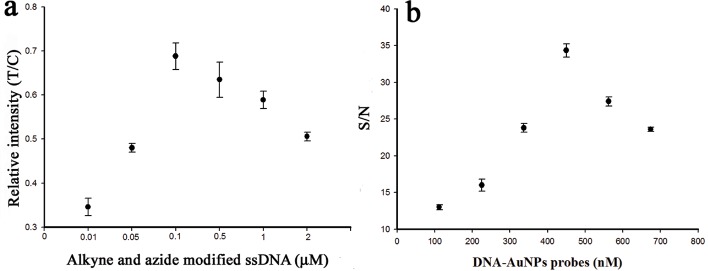
(a) Effect of the concentrations of the alkyne and azide modified ssDNA on the response of the SA-LFTS. (b) Effect of the combined concentration of DNA-AuNPs probes (probe 1 and probe 2) on the S/N ratio of the SA-LFTS method. The Cu^2+^ concentration was 10 μM. The error bars show the standard deviation (n = 3).

In the SA-LFTS, the azide modified DNA-A and alkyne modified biotinylated DNA-B acts as click reaction groups to form the new click ligation product, and the concentration of this product is closely correlated to the color intensity on the test line. Consequently, the concentrations of ssDNAs are important in the detection system. Different concentrations of the ssDNAs ranging from 0.01 μM to 2 μM, with a mole ratio of 1:1, were investigated in the presence of 10 μM Cu^2+^. The relative intensity (T/C) increased gradually as the DNA probe concentration increased up to 0.1 μM, and then decreased slowly at concentrations above 0.1 μM (Fig 3A). Excess biotinylated DNA-B would compete with the new click ligation products for reaction with streptavidin on the test line. Therefore, 0.1 μM was selected as the optimum concentration for both the azide and alkyne modified ssDNA.

### The concentration of DNA-AuNPs probes (probe 1 and 2) on the test strip

Accumulated AuNPs on the test and control lines were observed as red bands, that were used for visual detection of Cu^2+^. The concentration of the DNA-AuNPs probes (probe 1 and 2) on the two pads in the test strip would affect the sensitivity of this method. The effects of different concentration of DNA-AuNPs probes (ratio of probe 1:probe 2 = 1:1) on the signal-noise ratio (color intensity in test line determined in the presence and absence of Cu^2+^) in the presence of 10 μM Cu^2+^ were investigated (Fig 3B). The S/N ratio increased gradually as the concentration of the AuNPs-DNA probes increased from 112 to 450 nM. However, the S/N ratio decreased when the combined concentration of the AuNPs-DNA probes was above 450 nM because of increased nonspecific adsorption and a high background signal. Therefore, 450 nM of AuNPs-DNA probes was used for the test strip.

### Optimization of experimental parameters

The experimental parameters, including the click reaction time, the concentrations of THPTA and sodium ascorbate, the pH, and the running buffer, could all affect the analytical performance and were optimized in this study. A solution of 10 μM Cu^2+^ was used in the optimization experiments.

**Fig 4:**
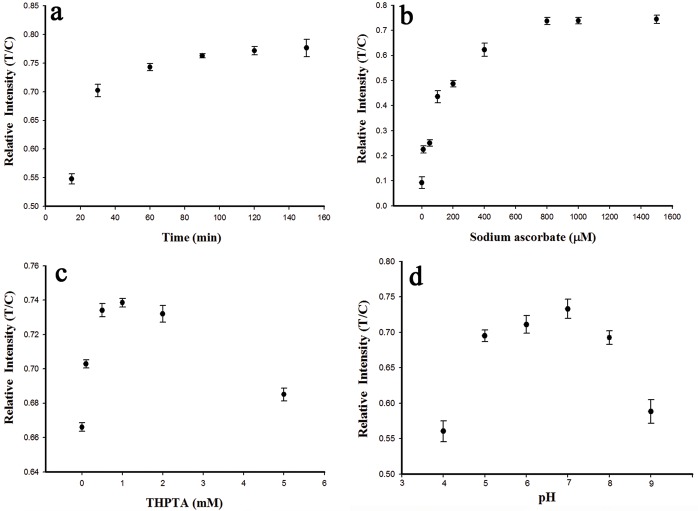
Effect of (a) click reaction time, (b) concentration of sodium ascorbate, (c) concentration of THPTA and (d) pH value. The concentration of Cu^2+^ used was 10 μM. The error bar shows the standard deviation (n = 3).

The color intensity of the test line directly depended on the yield of the click ligation ssDNA product, which in turn corresponded to the click reaction time for the Cu^+^-catalyzed cycloaddition. In 30 min, the click reaction was almost complete (90%) and the optical response only increased slightly after 30 min, reaching its maximum at 120 min (Fig 4A). Therefore, to achieve relatively rapid detection, 30 min was selected as the optimum click reaction time at little cost to the sensitivity.

Sodium ascorbate was used as to reduce the Cu^2+^, and an excess of this reagent increased the catalytic efficiency of Cu^2+^ [17,19]. The relationship between the optical response and different concentrations of sodium ascorbate (0 μM, 10 μM, 50 μM, 100 μM, 200 μM, 400 μM, 800 μM, 1000 μM, 1500 μM) was investigated. The optical response increased as the sodium ascorbate concentration increased up to 800 μM, after which it plateaued (Fig 4B). Thus, the optimum concentration of sodium ascorbate was 800 μM.

The effect of different concentrations of THPTA, which was used to increased Cu^+^ binding, on the relative intensity (T/C) was measured. A THPTA concentration of 1 mM gave the best sensing performance (Fig 4C).

The pH is known to have an important effect on cycloaddition reactions [23]. In the present study, the relative intensity (T/C) remained almost constant at pH values in the range of 5.0−8.0 and reached the highest value at pH 7.0 (Fig 4D), which was similar to a previous report [17]. The pH optimum was between 5.0 and 8.0 and a pH of 7.0 was used for the experiments described.

**Fig 5:**
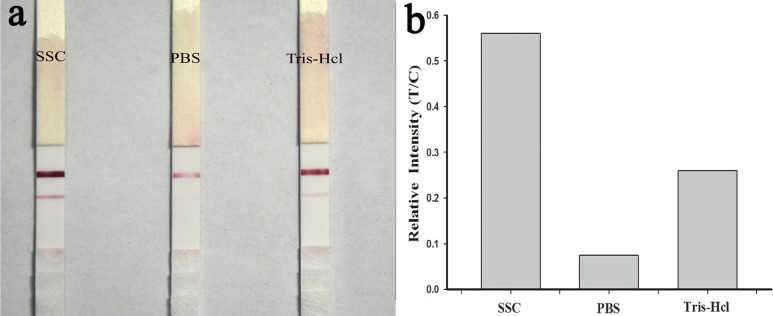
Photographs of Cu^2+^ sensing with different running buffers (4×SSC, 0.01 M PBS, and 0.01 M Tris-HCl).

The running buffer can greatly affect the sensitivity of SA-LFTS. Three different running buffers, including PBS, saline–sodium citrate (4×SSC), and Tris-HCl, were investigated. The 4×SSC buffer gave the best sensing performance (Fig 5), perhaps because of high hybridization between DNA-AuNPs probes in this buffer[20]. Consequently, 4×SSC (1% Tween-20) was selected as the running buffer for the test strip.

### Detection of Cu^2+^ under the optimum experimental conditions

**Fig 6:**
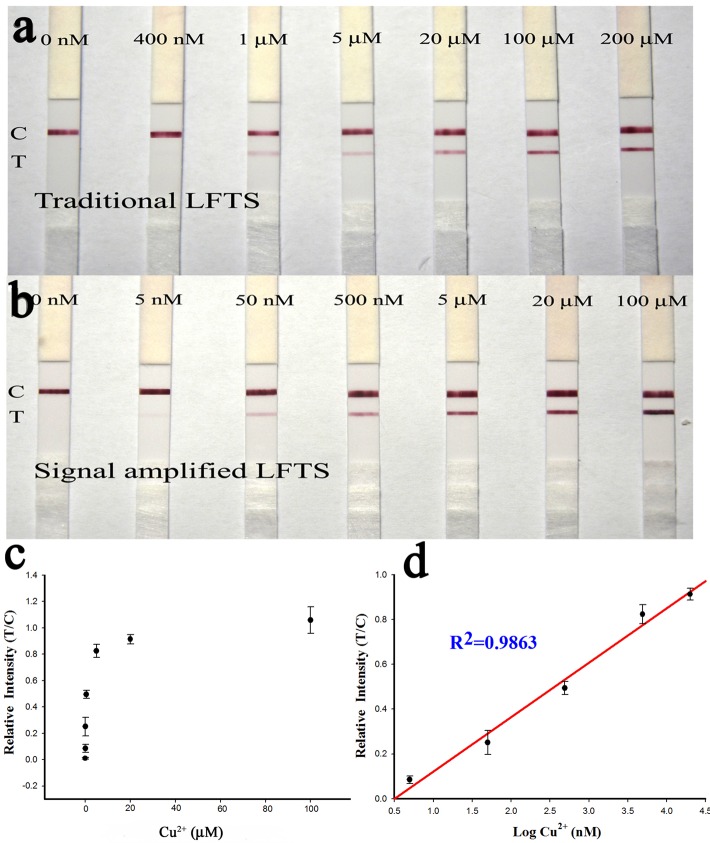
Results for Cu^2+^ sensing. Photographs of (a) traditional LFTS and (b) SA-LFTS. (c) Plot of the relative intensity (T/C) *vs* Cu^2+^ concentration. The color intensity were recorded with a strip reader. (d) Calibration curve of the relative intensity (T/C) *vs* the logarithm of Cu^2+^ concentration. Error bars show the standard deviations (n = 3).

The SA-LFTS was applied to detection of different concentrations of Cu^2+^ (5–100 μM) under the optimum conditions to investigate the sensitivity and dynamic range. Traditional LFTS was also used for Cu^2+^ detection, and the results with the two methods were compared to evaluate the signal amplification ability of the SA-LFTS. In the absence of Cu^2+^, there was no red band observed on the test line, indicating negligible nonspecific adsorption occurred (Fig 6B). In the presence of Cu^2+^, the color intensity of the red band on the test line increased with increasing Cu^2+^ concentration. The red band on the test line was quite visible even at 5 nM Cu^2+^, and this was defined as the limit of detection (LOD) for visual detection of Cu^2+^ without instrumentation. For quantitative analysis, the relative intensity (T/C) was recorded with a portable strip reader. The resulting calibration curve (Fig 6D) showed that the relative intensity were proportional to the logarithm of Cu^2+^ concentration in the range of 5 nM to 20 μM, and the LOD was 4.2 nM based on the 3σ rule, where σ is the relative standard deviation of the blank tests. By comparison, the visual detection range for conventional LFTS was from 400 nM to 200 μM, with a visual LOD of 400 nM (Fig 6A). These results show the LOD for visual detection of Cu^2+^ using the SA-LFTS was 80-times lower than that for traditional LFTS. Therefore, SA-LFTS provides good signal amplification. The detection limit of the developed method (4.2 nM) was four orders of magnitude lower than the US Environmental Protection Agency limit for Cu^2+^ in drinking water (20 μM).

### Specificity of the SA-LFTS

**Fig 7:**
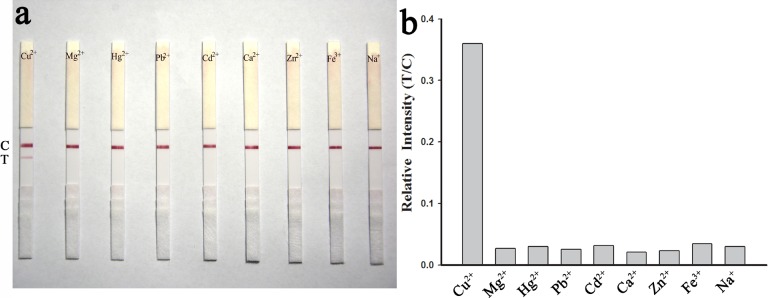
Specificity of the SA-LFTS for Cu^2+^ in the presence of competing metal ions. The concentration of Cu^2+^ was 100 nM and all the other ions were at 1000 nM.

The specificity of the SA-LFTS was investigated by comparing the color intensity of the test line with Cu^2+^ to that obtained with other metal ions, including Mg^2+^, Hg^2+^, Pb^2+^, Cd^2+^, Ca^2+^, Zn^2+^, Fe^3+^, Na^+^. Among the metal ions tested, only 100 nM Cu^2+^ produced a bright red band on the test line of the strip. No red band was observed on the test line for the other metal ions even at high concentration (1000 nM, Fig 7). These results suggest at these concentrations that SA-LFTS is specific for Cu^2+^, consistent with previous reports [17, 19].

**Fig 8:**
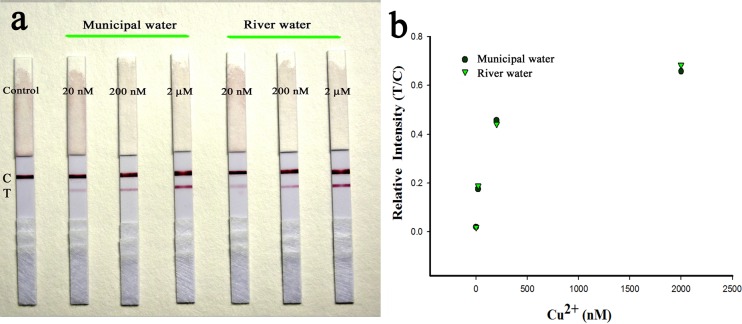
Sensing results of the SA-LFTS for detection of Cu^2+^ in spiked municipal water and river water samples.

The practical application of our proposed SA-LFTS system was demonstrated by applying it to detect Cu^2+^ in municipal water and river water samples spiked with various concentrations of Cu^2+^ (20 nM, 200 nM and 2 μM) (Fig 8**)**. The recoveries were between 92.30 and 106.52% (Table 1). These results were confirmed by comparison with those obtained by inductively coupled plasma mass spectrometry, and this comparison indicated the developed method has good accuracy for Cu^2+^. These results demonstrate the potential of the developed method for the rapid detection of Cu^2+^ in water samples.

**Table 1:** Comparison of SA-LFTS and ICP-MS results for water samples spiked with Cu^2+^ at various concentrations.

Sample	Spiked Cu^2+^ (nM)	The proposed method (SA-LFTS)			ICP-MS		
		Measured±SD^a^ (nM)	Recovery^b^ (%)	CV^c^ (%)	Measured±SD^a^ (nM)	Recovery^b^ (%)	CV^c^ (%)
Municipal water	20	19.50±1.19	97.50	6.10	20.80±1.24	104.00	5.96
	200	203.27±5.54	101.64	2.72	209.50±4.63	104.75	2.21
	2000	2130.42±45.12	106.52	2.12	2080.67±49.17	104.03	2.36
River water	20	18.46±1.76	92.30	9.53	19.41±1.05	97.05	5.40
	200	204.86±4.64	102.43	2.26	205.70±3.70	102.85	1.80
	2000	2107.32±64.23	105.36	3.05	2010.47±74.12	100.52	3.69

^a^ Mean measured concentration of three replicates ± standard deviation.

^b^ Mean recovery (%) = 100 × (C_mean measured_/C_added_).

^c^ Coefficient of variation of three determinations.

## Conclusions

In summary, we successfully developed an enhanced SA-LFTS for the visual detection of Cu^2+^ in aqueous solution. The SA-LFTS method was based on Cu^+^-catalyzed click chemistry and ssDNA hybridization-induced signal amplification. The lateral flow strip assay was sensitive for Cu^2+^ with an apparent LOD of ~4 nM. The LOD is much lower than the US Environmental Protection Agency limit for Cu^2+^ in drink water (20 μM). When the SA-LFTS was applied to the monitoring of Cu^2+^ spiked into municipal and river water samples, it showed good recovery and accuracy. Importantly, the generated signals could were strong enough to be observed by the naked eye. The performance of the SA-LFTS method was compared in terms of time, LOD, and instrumental requirements to other Cu^+^-promoted click chemistry based methods that have been described previously for the determination of Cu^2+^ (Table 2). We expect that the developed SA-LFTS will be a promising new tool for trace Cu^2+^ analyses, and will find wide application in field-based testing.

**Table 2:** Comparison of different Cu^+^-promoted click chemistry methods for the detection of Cu^2+^.

Output signal	Time (min)	LOD^a^	Instrumental required	Ref.
Colorimetric	>120	50 μM	No	[15]
Colorimetric	60	250 nM	No	[17]
Gold nanoparticles accumulated signal	60	100 nM	No	[19]
Colorimetric	140	5.9 nM	No	[24]
Commercial glucometer	>120	10 nM	Yes	[25]
Fluorescence	20	2 nM	Yes	[26]
Fluorescence	120	290 nM	Yes	[27]
Fluorescence	30	58 nM	Yes	[28]
Gold nanoparticles accumulated signal	40	5 nM	No	This work

^a^ Limit of detection.
